# A Novel Reversed U Curve Method to Facilitate Ethanol Infusion into the Vein of Marshall in Atrial Fibrillation: A Single-Center Case Series Study

**DOI:** 10.3390/jcdd13060246

**Published:** 2026-06-03

**Authors:** Qinchao Wu, Xu Liu, Zhuo Liang, Yanguang Li, Qiaoyuan Li, Sixian Weng, Lili Wang, Yijie Liu, Zhipeng Hu, Jiawei Zhang, Ran Xiong, Yunlong Wang

**Affiliations:** Department of Cardiology, Beijing Anzhen Hospital Affiliated to Capital Medical University, No. 2 Anzhen Road, Chaoyang District, Beijing 100029, China

**Keywords:** atrial fibrillation, ethanol infusion, vein of Marshall, venography

## Abstract

Background: Ethanol infusion into the vein of Marshall (EI-VOM) is widely used to facilitate atrial fibrillation ablation and block of the mitral isthmus. However, it cannot be completed in some anatomically challenging cases. This study aimed to introduce and evaluate a novel reversed U curve method of EI-VOM. Methods: This case-series study enrolled consecutive patients with atrial fibrillation or atrial flutter who were scheduled for EI-VOM. When VOM venography was successfully performed, the conventional EI-VOM method was attempted first. If this approach failed or took ≥20 min, the novel method was applied. The success rate, complications, and applicable anatomical conditions of the new method were summarized. Results: Of the 205 patients enrolled in this study, the novel method was applied to 45 patients, and technical success was achieved in 42 patients (93.3%). Among the patients who underwent the novel method, twenty-four (53.3%) had a long cavotricuspid isthmus, nineteen (42.2%) had a VOM ostium close to the coronary sinus ostium, and sixteen (35.6%) had a prominent Eustachian ridge. The total mean procedure time of EI-VOM using the novel method was 30.00 ± 4.5 min. Acute bidirectional mitral isthmus block was achieved in 40 cases (88.9%), and the mean ethanol volume injected was 8.21 ± 1.5 mL. No serious in-hospital complications were documented in patients treated with the novel method. Conclusions: In this single-center case series, the reversed U curve method appeared feasible as a femoral bailout strategy for EI-VOM in selected anatomically challenging cases. Further prospective, multicenter studies involving multiple operators are required to confirm its reproducibility, efficacy, and safety over conventional approaches.

## 1. Introduction

The ligament of Marshall, known as the epicardial connection between the left atria, is richly innervated by sympathetic and parasympathetic nerve fibers that play important roles in the initiation and perpetuation of atrial fibrillation (AF) [1,2]. Ethanol infusion into the vein of Marshall (EI-VOM) can eliminate AF triggers, enhance atrial denervation, simplify electrical isolation of the left pulmonary vein, and facilitate mitral isthmus block [3]. Increasing clinical evidence has demonstrated the value of EI-VOM in the ablation of persistent AF (peAF) [4,5,6,7].

Nevertheless, EI-VOM cannot be achieved in all cases due to the complex anatomy of the VOM and its adjacent structures. In a previous report, EI-VOM was successfully performed in 80–90% of patients [8]. In early clinical practice, EI-VOM was performed using the superior vena cava (SVC) approach via the right internal jugular vein [9]. The inferior vena cava approach via the femoral vein is widely used in clinical practice, because it may reduce additional puncture-related risks and can be integrated into the femoral ablation workflow [10]. However, EI-VOM is difficult to accomplish in some patients via the inferior vena cava approach due to specific anatomical structures. Based on our experience, we developed a novel reversed U curve method to simulate the SVC approach via the femoral vein using a steerable sheath as a femoral bailout strategy for EI-VOM.

## 2. Methods

### 2.1. Study Population

**Figure 1:**
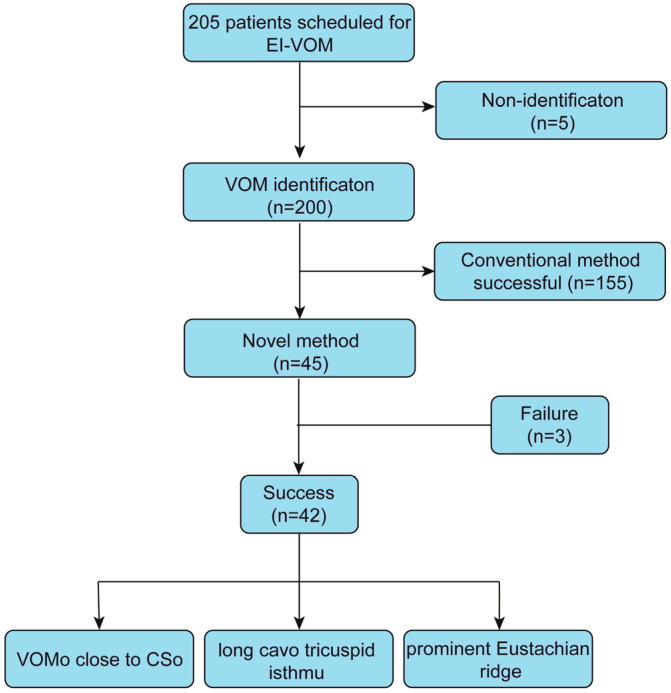
Flowchart of patient enrollment. Among the 205 patients scheduled for EI-VOM, 5 patients had non-identification of the VOM. Among the remaining 200 patients with identifiable VOMs, the conventional femoral approach was successful in 155 patients, and the reversed U curve method was attempted in 45 patients after failure or prolonged attempts with the conventional femoral approach. Technical success with the reversed U curve method was achieved in 42 patients. EI-VOM, ethanol infusion into the vein of Marshall; VOMo, VOM ostium; CSo, coronary sinus ostium.

Between July 2024 and November 2025, consecutive patients with AF or atrial flutter (AFL) scheduled for radiofrequency catheter ablation and EI-VOM at Beijing Anzhen Hospital were enrolled. When VOM venography was successfully performed, the conventional EI-VOM method was attempted first, and if it failed or took ≥20 min, a novel reversed U curve method was applied [11]. The success of EI-VOM was defined as successful guidewire and balloon cannulation of the VOM, and completion of ethanol delivery. The 20-min threshold was used as a pragmatic procedural cutoff to avoid prolonged manipulation, excessive fluoroscopy, and potential VOM injury. Patients who underwent EI-VOM using the novel method were included in the final analyses. A flowchart of patient enrollment is shown in Figure 1. Informed consent was obtained from all patients. The study was conducted in accordance with the principles of the Declaration of Helsinki. The Institutional Ethics Committee of Beijing Anzhen Hospital approved the study protocol. A flowchart of patient enrollment is presented in Figure 1.

### 2.2. Procedural Strategy

Transesophageal echocardiography, enhanced computed tomography, or intra-cardiac echocardiography (Soundstar, Biosense Webster, Irvine, CA, USA, or Viewflex Xtra, Abbott, MN, USA) was performed to exclude intra-cardiac thrombi in all patients. After placing a steerable electrode catheter within the coronary sinus (CS) from the left femoral vein, a transseptal puncture was performed under fluoroscopic guidance, and an SL1 long sheath or a steerable sheath (Agilis NxT; Abbott, MN, USA or Navigo; Synaptic Medical, Beijing, China) from the right femoral vein was introduced into the left atrium. Heparin (100 IU/kg) was administered to achieve an activated clotting time > 300 s. Subsequent boluses of heparin were administered every 20–30 min, if necessary. The ablation procedure was performed under conscious sedation using the CARTO 3 (Biosense Webster, Irvine, CA, USA) or Ensite (Abbott, MN, USA) mapping systems. For patients with peAF, EI-VOM was planned when at least one of the following characteristics was present: long-standing peAF, left atrial volume > 150 mL, or left atrial diameter > 43 mm. The peAF ablation strategy involved EI-VOM, pulmonary vein isolation, and linear ablation of the roof, mitral, and cavotricuspid isthmus (CTI). Redo ablation procedures included EI-VOM for one of these three reasons: (1) treatment of clinical tachycardia if the VOM bundle was potentially involved; (2) blocking the mitral line, if not yet achieved; or (3) as an adjunct therapy for peAF beyond the initial lesion set created during the first procedure. For patients with atrial flutter, EI-VOM was performed when intra-procedural mapping suggested mitral isthmus-dependent flutter.

### 2.3. EI-VOM

**Figure 2:**
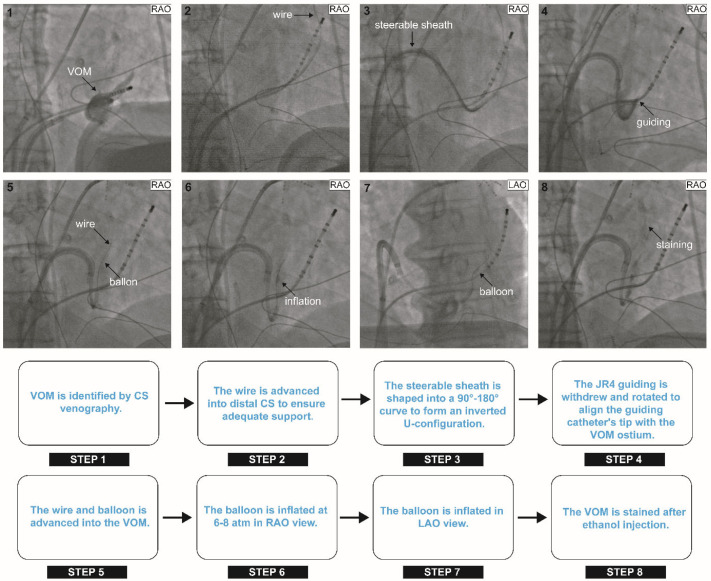
Detailed procedure process of the novel reversed U curve method. VOM, vein of Marshall; CS, coronary sinus; RAO, right anterior oblique; LAO, left anterior oblique.

First, EI-VOM was performed using the conventional method. The CS was cannulated by a 6 F Judkins right guiding catheter inside the SL1 long sheath or steerable sheath. CS venography was performed by Judkins right guiding catheter to identify the presence of the VOM. If present, an over-the-wire angioplasty balloon (1.5–2.0 mm × 6–8 mm; Boston Scientific, Marlborough, MA, USA) preloaded with guidewire was advanced into the VOM, and 5–10 mL of 95% ethanol was injected into the VOM over 5 min, with selective VOM angiography repeated each time to confirm balloon stability and staining of the VOM territory before ethanol delivery. If the conventional method failed or the conventional method required ≥20 min, a novel reversed U curve method to simulate the SVC approach via the femoral vein using a steerable sheath was attempted. Figure 2 shows the procedural process step by step: (1) the VOM is identified by CS venography; (2) the wire is advanced to the distal CS to ensure adequate support; (3) the steerable sheath is shaped into a 90–180° curve to form an inverted U-configuration with the wire providing support; (4) the Judkins right guiding catheter is withdrawn and rotated to align the guiding catheter’s tip with the VOM ostium; (5) the wire and balloon are advanced into the VOM; (6) the balloon is inflated at 6–8 atm; (7) the VOM is stained after ethanol injection. The total procedural time was defined as the total time from the initial attempt using the conventional femoral vein approach to the final completion or abandonment of EI-VOM. To ensure procedural consistency, all reversed U curve procedures were performed by a single experienced operator, Yunlong Wang. Dr. Wang is a Chief Physician in the Third Arrhythmia Ward of Beijing Anzhen Hospital and a senior electrophysiologist. He performs more than 1000 electrophysiological procedures annually, including more than 300 EI-VOM procedures per year.

### 2.4. Measurement

The anatomical structure of the VOM and isthmus of the tricuspid valve were analyzed using radiography. The length of the CTI, the vertical distance from the VOM ostium (VOMo) to the CS ostium (CSo), and the VOMo diameter, were measured from the right anterior oblique (RAO) view. The horizontal vertical distance from the VOMo to the CSo in the left anterior oblique (LAO) and RAO views was recorded. In this study, CTI length was operationally defined as the horizontal distance from the inferior vena cava region to the CSo in the RAO view. Because no universally accepted angiographic cutoff exists for defining long CTI in EI-VOM procedures, long CTI was defined in this study as a CTI distance > 42 mm, based on the measured data from the present cohort. VOMo close to the CSo was defined as a VOMo-to-CSo distance ≤ 8 mm measured in the RAO view, and ≤18 mm in the LAO view. Prominent Eustachian ridge was defined as an intra-procedural fluoroscopic and operational finding. A prominent Eustachian ridge was considered present when, in the RAO view, the sheath or guiding catheter showed marked abnormal bulging, deflection, or instability at the inferior vena cava–right atrium junction, thereby interfering with smooth catheter advancement, coronary sinus engagement, or stable guiding-catheter support. The external diameter of the 8.5 F long sheath was used as a reference for the unified measurement. A specific diagram of the measurement is presented in Supplementary Figure S1. The measurements were independently performed offline by Qinchao Wu, Xu Liu, and Yanguang Li, who were blinded to the procedural outcome at the time of measurement. The final values were averaged across the three measurements. In cases of significant discrepancies, remeasurement was performed after discussion.

### 2.5. Complications

Safety endpoints mainly focused on peri-procedural and in-hospital complications. All patients underwent at least one transthoracic echocardiographic examination within 24 h after the procedure and repeated electrocardiographic examinations during hospitalization. Procedure-related complications were defined as CS dissection, VOM dissection, cardiac tamponade, pericarditis, vascular complications, or atrioesophageal fistula.

### 2.6. Statistical Analysis

The baseline characteristics were presented as the mean (standard deviation [SD]), median [interquartile range (IQR)], or number (percentage), as appropriate. All statistical analyses were conducted using the Statistical Package for the Social Sciences (SPSS) version 27.0 (IBM Corp., Armonk, NY, USA).

## 3. Results

### 3.1. Baseline Characteristics

In total, the novel reversed U method was applied in 45 patients. The mean age was 59.24 ± 10.4 years, and 71.1% were male. In the overall population, paroxysmal AF was present in 8.9%, persistent AF in 80.0%, and AFL in 11.1% of patients. The mean left atrial diameter and left atrial volume were 45.24 ± 4.5 mm and 147.27 ± 26.7 mL, respectively. The total mean procedural time of EI-VOM was 30.00 ± 4.5 min, and the longest total EI-VOM attempt was 51 min. Baseline characteristics of the study population are summarized in Table 1.

### 3.2. EI-VOM Results of the Novel Method

**Figure 3:**
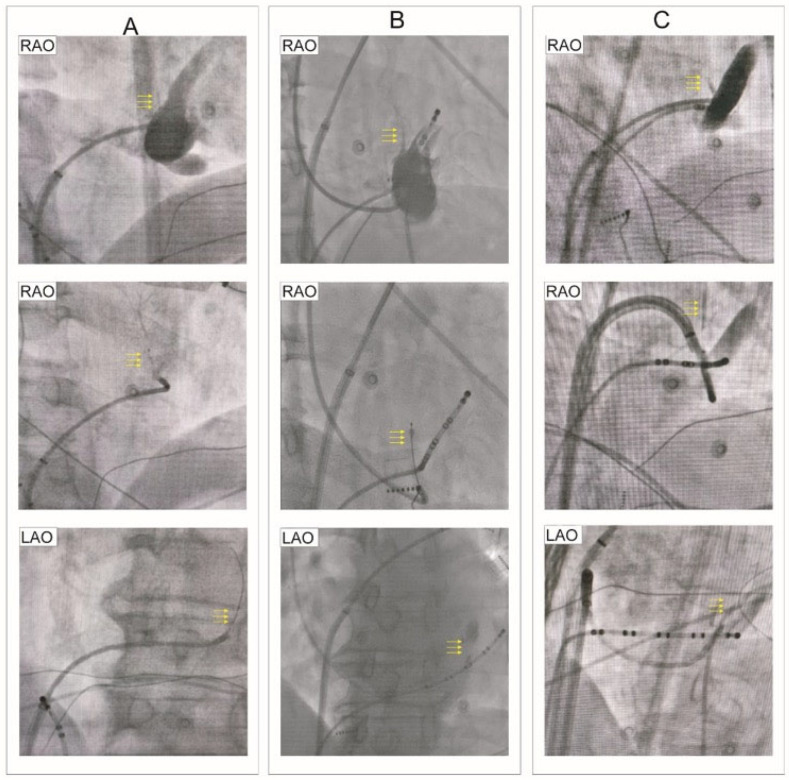
Examples for performing EI-VOM using different methods (**A**) EI-VOM by conventional method; (**B**) EI-VOM by SVC approach via jugular vein; (**C**) EI-VOM by simulating the SVC approach via femoral vein using steerable sheath. The yellow arrow indicates the VOM. RAO, right anterior oblique; LAO, left anterior oblique.

**Figure 4:**
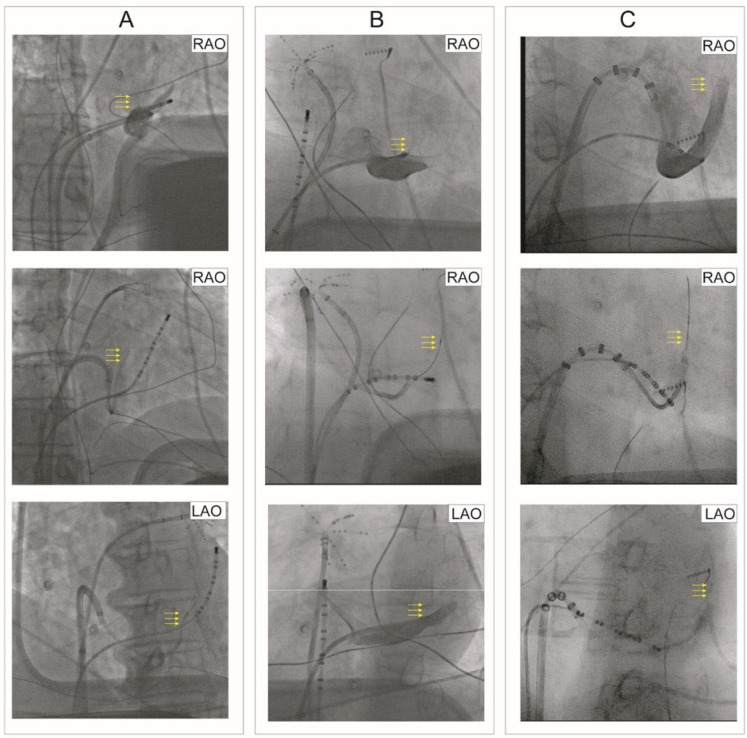
Examples of the novel reversed U curve method. (**A**) Vein of Marshall ostium close to the coronary sinus ostium; (**B**) long cavotricuspid isthmus; (**C**) prominent Eustachian ridge. The yellow arrow indicates the VOM. RAO, right anterior oblique; LAO, left anterior oblique.

**Figure 5:**
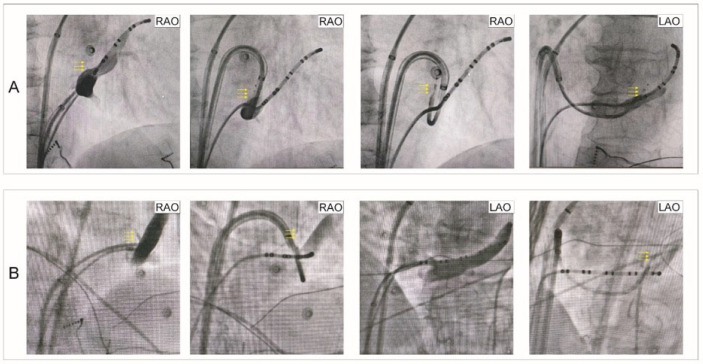
Two scenarios of the VOM ostium appearing close to the CSo in the RAO view. (**A**) The VOM close to the CSo in both the RAO and LAO views. (**B**) The VOM close to the CSo in the RAO view but actually not close in the LAO view. The yellow arrow indicates the VOM. RAO, right anterior oblique; LAO, left anterior oblique.

Among the 205 patients scheduled for EI-VOM, the VOM could not be identified in 5 patients. In the remaining 200 patients, the conventional method was successful in 155 patients, and the reversed U curve method was attempted in 45 patients because of conventional-method failure or procedure time ≥ 20 min. The reversed U curve method achieved technical success in 42 patients (42/45, 93.3%). The method failed in three cases because of a small VOMo diameter and/or an extremely prominent Eustachian ridge. Acute bidirectional mitral isthmus block was achieved in 40 cases (88.9%), and the mean ethanol volume injected was 8.21 ± 1.5 mL. Based on imaging characteristics, the anatomical scenarios suitable for this approach were classified into three overlapping categories (Table 2). Figure 3 illustrates the differences between EI-VOM by the conventional method, the SVC approach via the jugular vein, and the reversed U curve method simulating the SVC approach from the femoral vein. Figure 4 presents three scenarios in which the reversed U curve method can be applied. Among the patients who underwent the reversed U curve method, the most common associated condition was a long CTI (24/45, 53.3%). Nineteen patients (42.2%) had a VOMo close to the CSo, and 16 (35.6%) had a prominent Eustachian ridge. Because these categories were overlapping, 15 patients (33.3%) exhibited two or more of these anatomical features, whereas 7 patients (15.6%) had none of the predefined features. In some patients without these predefined features, the VOMo appeared close to the CSo in the RAO view but was not actually close in the LAO view because of projectional compression (Figure 5).

### 3.3. Complications

In total, VOM dissection occurred in 1 patient using the novel method (1/45, 2.2%). No CS dissection, cardiac tamponade, pericarditis, vascular complications, and atrioesophageal fistula occurred in the patients who underwent the novel method.

## 4. Discussion

This case-series study introduced a novel reversed U curve method that simulated the SVC approach via the femoral vein using a steerable sheath in patients with specific anatomical structures. Scenarios suitable for the novel method included VOMo close to the CSo, long CTI, and prominent Eustachian ridge. The novel method may serve as a femoral bailout strategy in selected cases in which the conventional femoral approach is unsuccessful or technically difficult, which requires further verification in future studies.

Initially, EI-VOM was performed via the jugular vein using the SVC approach [9]. With advances in clinical experience, the inferior vena cava approach via the femoral vein has now been widely used for EI-VOM. The femoral vein approach may offer several practical advantages, including lower radiation exposure, fewer puncture-related complications, and the ability to use steerable sheaths [10]. However, due to anatomical constraints, performing EI-VOM via the femoral vein approach can be challenging [12]. Therefore, this study aimed to evaluate the feasibility of the reversed U curve method as a femoral bailout strategy in anatomically challenging EI-VOM cases.

In this study, the reversed U curve method was applied in 45 patients after unsuccessful or prolonged conventional femoral attempts and achieved technical success in 42 patients (93.3%). This finding suggests that the reversed U curve method may be feasible as a bailout strategy in selected anatomically challenging cases. However, because this study was not designed as a comparative cohort study, the observed success rate should be interpreted as a descriptive feasibility finding rather than evidence of superiority over the conventional femoral approach. Three scenarios were identified suitable for applying the novel approach: VOMo close to the CSo, long CTI, and prominent Eustachian ridge.

When the VOM is located close to the CSo, the guiding catheter tends to slip out of the CS, and maintaining proper wire-VOM coaxiality is often challenging. Forced manipulation may lead to VOM dissection and even VOM perforation, compromising the efficacy of EI-VOM. We expanded the maneuvering space for the guiding catheter by simulating the SVC approach. This technique enhances stability by anchoring the guiding catheter against the CS floor while simultaneously improving wire coaxiality. Actually, the proportion of these patients with closely positioned orifices may have been overestimated in this study. In some cases, the VOM was not visualized in the LAO view. We observed that in certain cases that the VOMo appeared close to the CSo in the RAO view and the actual distance was not short in the LAO view, which was caused by distance compression in the RAO view. In such scenarios, if the VOMo can be fully visualized in the LAO view, the conventional method can be performed in the LAO view. If VOMo cannot be clearly exposed in the LAO view, the novel method may be attempted in the RAO view.

In this study, we found that a long cavotricuspid isthmus was the most common coexisting anatomical feature in AF or AFL patients. A long tricuspid isthmus increases guiding catheter tension, hinders CS cannulation, and compromises EI-VOM maneuverability, thus increasing procedural difficulty. While catheter shaping or steerable sheath utilization can mitigate these challenges, coexisting anatomical variations, including a prominent Eustachian ridge or short distance from the VOM to the CSo, may render the procedure exceptionally demanding. The simulated SVC-like approach may be useful in such complex scenarios.

The Eustachian ridge presents a significant obstacle during EI-VOM. A prominent Eustachian ridge can impede the sheath and guide catheter advancement into the CS and VOM. The guiding catheter tends to form a reverse S shape, increasing catheter instability and making antegrade–retrograde manipulation difficult. By simulating the SVC approach, this technique bypasses the Eustachian ridge obstruction, enhancing guiding catheter stability and maneuverability and thereby facilitating successful EI-VOM.

In terms of safety, no procedure-related complications, such as pericarditis, cardiac tamponade, clinically significant pericardial effusion, or unintended left atrial appendage isolation, were observed in patients treated with the reversed U curve method. VOM dissection occurred in one patient (1/45, 2.2%) by novel method and 12 patients (12/155, 7.7%) by conventional method. However, no formal statistical comparison was performed, and these data should not be interpreted as evidence that the novel method reduces the risk of VOM dissection. The low incidence observed in the reversed U curve group may be partly related to improved wire–VOM coaxiality and reduced direct engagement of the VOM ostium, but this hypothesis requires validation in comparative studies. With the SVC approach, guidewire advancement into the VOM can sometimes be achieved even when the catheter tip is not perfectly aligned with the VOM ostium, thereby reducing the need for forceful ostial engagement. In contrast, during the conventional femoral approach using a Judkins-type catheter, the catheter tip may directly press against the VOM ostium, especially when the VOM ostium is close to the coronary sinus ostium or when the catheter–VOM angle is unfavorable, potentially increasing the risk of VOM dissection. The reversed U curve method was designed to simulate some of the mechanical advantages of the SVC approach while preserving femoral venous access. By reshaping the sheath–catheter system into a reversed U configuration, this technique may improve the support angle and wire–VOM coaxiality, allowing the guidewire rather than the catheter tip to enter the VOM first. Nevertheless, because this study did not directly compare the reversed U curve method with either the conventional method or the SVC approach, we cannot conclude that this technique is safer than the conventional method or SVC approach. The safety assessment mainly focused on common peri-procedural and in-hospital complications. Thus, rare or delayed complications require further evaluation in larger prospective studies with systematic safety monitoring.

It should be noted that the femoral approach should not be regarded as definitively superior to the SVC approach, because no direct comparative study has evaluated these two approaches. In anatomically difficult cases, the SVC approach may have potential advantages. The reversed U curve method was designed to partially overcome the limitation of femoral approach by reshaping the guiding system and improving the angle of support and wire–VOM coaxiality. Therefore, this technique may serve as a femoral bailout strategy in selected cases in which the conventional femoral approach is unsuccessful or technically difficult. In the present study, conversion to the SVC approach was not systematically attempted in the three patients in whom both the conventional femoral approach and the reversed U curve method failed. Therefore, we cannot determine whether the SVC approach would have been successful in these cases. Future studies directly comparing the conventional femoral approach, the reversed U curve method, and the SVC approach are needed to clarify the optimal bailout strategy for different anatomical scenarios.

Another practical bailout strategy for VOM cannulation is the double-wire technique [11]. When the VOM ostium is close to the CSo but the guiding catheter can still maintain reasonable support within the CS, the double-wire technique may help stabilize the system and facilitate VOM entry with limited additional sheath manipulation. In contrast, the reversed U curve method may be preferable when the main limitation is an unfavorable femoral approach angle, catheter slippage from the CS, a long CTI, or a prominent Eustachian ridge that prevents coaxial alignment. The double-wire technique is technically simple and uses familiar femoral access, but it may not overcome severe catheter instability or inadequate approach angle. The reversed U curve method provides a more SVC-like approach angle through the femoral route, but it requires a steerable sheath and careful manipulation to avoid excessive tension. The optimal choice should be individualized according to VOMo location, CS anatomy, catheter stability, and operator experience.

## 5. Conclusions

Conventional EI-VOM method can be challenging to accomplish in cases with specific anatomical structures, including a long CTI, VOMo close to the CSo, and prominent Eustachian ridge. In this single-center case series, the reversed U curve technique appeared feasible as a bailout strategy for EI-VOM in selected anatomically challenging cases. Further prospective, multicenter studies involving multiple operators are required to confirm its reproducibility, safety, and incremental value over conventional approaches.

## 6. Limitations

Several limitations should be acknowledged. First, this was a single-center observational case series, and all reversed U curve procedures were performed by a single experienced operator. Therefore, the reproducibility of this technique remains unknown. Second, the safety assessment mainly focused on peri-procedural and in-hospital complications in this study. Thus, rare or delayed complications require further evaluation in larger prospective studies with systematic safety monitoring. Third, this study did not include a formal comparison between the conventional femoral approach, the reversed U curve method, and the SVC approach. Prospective multicenter studies involving multiple operators are needed to compare the efficacy and safety between different methods of EI-VOM. Fourth, the angiographic cut-off values for defining long CTI and VOMo–CSo proximity were derived from the observed distribution in this single-center cohort. Moreover, the diagnosis of a prominent Eustachian ridge was based on fluoroscopic appearance rather than quantitative imaging. Future larger, multicenter studies with predefined, standardized criteria are needed to validate these anatomical predictors.

## Figures and Tables

**Table 1:** Baseline characteristics.

Characteristics	Novel Method (n = 45)
Age, mean (SD), years	59.24 (10.4)
Gender, n (%)	
Male	32 (71.1)
Female	13 (28.9)
BMI, mean (SD), kg/m^2^	28.01 (3.8)
Medical history, n (%)	
Hypertension	27 (60.0)
Diabetes mellitus	15 (33.3)
Stroke	4 (8.9)
Heart failure	18 (40.0)
Coronary artery disease	3 (6.7)
Type of arrhythmias, n (%)	
Persistent AF	36 (80.0)
Paroxysmal AF	4 (8.9)
Atrial flutter	5 (11.1)
CHA_2_DS_2_-VASc score	2.11 (1.5)
Left atrial diameter, mean (SD), mm	45.24 (4.5)
Right atrial diameter, mean (SD), mm	44.18 (5.8)
Left atrial volume, mean (SD), mL	147.27 (26.7)
LVEF, mean (SD), %	60.29 (7.5)
Length of CTI, mean (SD), mm	40.92 (8.9)
Horizontal distance from VOMo to CSo, mean (SD), mm	9.07 (6.0)
Diameter of VOMo, mean (SD), mm	1.53 (0.7)
Total procedural time of EI-VOM, mean (SD), min	30.00 (4.5)
Ethanol volume, mean (SD), mL	8.21 (1.5)
Acute bidirectional mitral isthmus block, n (%)	40 (88.9)
Balloon size, n (%)	
1.5 mm	27 (60.0)
2.0 mm	18 (40.0)

BMI: body mass index; AF: atrial fibrillation; SD: standard deviation; LVEF: left ventricular ejection fraction; CHA_2_DS_2_-VASc: congestive heart failure, hypertension, age > 75 years (doubled), diabetes, stroke/transient ischemic attack/thromboembolism (doubled), vascular disease (prior myocardial infarction, peripheral artery disease, or aortic plaque), age 65–75 years, sex category (female); CTI: cavotricuspid isthmus; CSo: coronary sinus ostium; VOMo: ostium of vein of Marshall.

**Table 2:** Angiography results.

	Length of CTI	Distance from VOMo to CSo		Diameter of VOMo
	RAO View	RAO View	LAO View	RAO View
All patients (N = 45)	41.41 ± 8.51	8.29 ± 4.40	21.86 ± 10.19	1.58 ± 0.66
Group A (N = 19)	39.48 ± 9.19	5.62 ± 2.52	13.16 ± 4.59	1.78 ± 0.62
Group B (N = 24)	47.74 ± 4.33	10.28 ± 7.21	23.85 ± 10.38	1.59 ± 0.67
Group C (N = 16)	44.28 ± 6.87	10.48 ± 8.45	23.42 ± 12.67	1.40 ± 0.65

The anatomical categories were overlapping rather than mutually exclusive. Group A: VOMo close to CSo, defined as VOMo-to-CSo distance ≤ 8 mm in the RAO view and ≤18 mm in the LAO view; Group B: long cavotricuspid isthmus, defined as CTI distance > 42 mm in the RAO view; Group C: prominent Eustachian ridge, defined as an intra-procedural fluoroscopic and operational finding. CTI: cavotricuspid isthmus; CSo: coronary sinus ostium; VOMo: ostium of vein of Marshall; RAO: right anterior oblique; LAO: left anterior oblique.

## Data Availability

All data included in this study are available upon request by contact with the corresponding author.

## Supplementary Materials

The following supporting information can be downloaded at: https://www.mdpi.com/article/10.3390/jcdd13060246/s1, Figure S1: Diagram of the measurement.
