# Supplementary material for: A Novel Reversed U Curve Method to Facilitate Ethanol Infusion into the Vein of Marshall in Atrial Fibrillation: A Single-Center Case Series Study
Source: J Cardiovasc Dev Dis. 2026 Jun 3;13(6):246. doi: 10.3390/jcdd13060246 (PMC13300134; doi:10.3390/jcdd13060246)
Supplement: Supplementary file 1 [file jcdd-13-00246-s001.zip › jcdd-4288836-supplementary.pdf]

Supplementary Figure S1. Diagram of the measurement

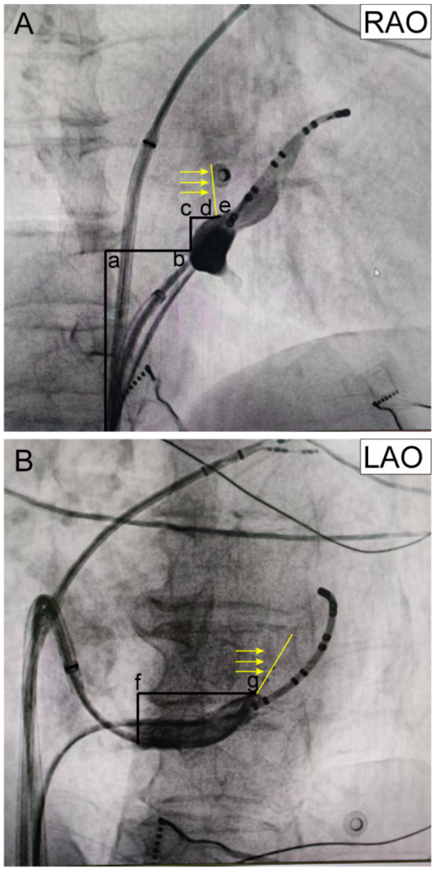

In Figure S1A, the yellow arrow indicates the VOM, ab indicates the length of cavo tricuspid isthmus, bc, cd indicates the vertical and horizontal distance from VOMo to CSo in the RAO view, de indicates the diameter of VOMo. In Figure S1B, ef indicates the horizontal distance from the VOMo to the CSo in the LAO view. VOM: vein of marshall; CSo: coronary sinus ostium; VOMo: ostium of vein of marshall; RAO: right anterior oblique; LAO: left anterior oblique.
